# Rare, common, alien and native species follow different rules in an understory plant community

**DOI:** 10.1002/ece3.8734

**Published:** 2022-03-21

**Authors:** Sarah Reeve, David C. Deane, Chris McGrannachan, Gillis Horner, Cang Hui, Melodie McGeoch

**Affiliations:** ^1^ 2541 School of Biological Sciences Monash University Melbourne Victoria Australia; ^2^ 2541 School of Life Sciences Department of Ecology, Evolution and Environment La Trobe University Bundoora Victoria Australia; ^3^ Manaaki Whenua—Landcare Research Auckland New Zealand; ^4^ School of Ecosystem and Forest Sciences University of Melbourne Richmond Victoria Australia; ^5^ Centre for Invasion Biology Department of Mathematical Sciences Stellenbosch University Matieland South Africa; ^6^ Biodiversity Informatics Unit African Institute for Mathematical Sciences Cape Town South Africa; ^7^ 549434 International Initiative for Theoretical Ecology London UK

**Keywords:** biological invasion, compositional turnover, distance decay, environmental filtering, multispecies introduction, understory plant community structure, zeta diversity

## Abstract

Biological invasions are a leading threat to biodiversity globally. Increasingly, ecosystems experience multiple introductions, which can have significant effects on patterns of diversity. The way these communities assemble will depend partly on whether rare and common alien species respond to environmental predictors in the same manner as rare and common native species, but this is not well understood. To examine this question across four national parks in south‐eastern Australia, we sampled the understory plant community of eucalypt‐dominated dry forest subject to multiple plant introductions. The drivers of diversity and turnover in alien and native species of contrasting frequency of occurrence (low, intermediate, and high) were each tested individually. We found alien species diversity and turnover were both strongly associated with abiotic conditions (e.g., soil pH), while distance had little influence because of the greater extent of occurrence and more homogeneous composition of common aliens. In contrast, native species diversity was not associated with abiotic conditions and their turnover was as strongly influenced by distance as by abiotic conditions. In both alien and native species, however, the most important predictors of turnover changed with frequency of occurrence. Although local coexistence appears to be facilitated by life history trade‐offs, species richness of aliens and natives was negatively correlated and native species might face greater competition in areas with more neutral soils (e.g., pH > ~5.5) where alien richness and relative frequency were both highest. We conclude that diversity and turnover in the generally more widespread alien species are mainly driven by species sorting along an environmental gradient associated with pH and nutrient availability, whereas turnover of native species is driven by more neutral processes associated with dispersal limitation. We show alien and native plant species respond to different environmental factors, as do rare and common species within each component.

## Graphical abstract

Many parks and forests have experienced centuries of multispecies invasions. Using a multi‐site turnover metric, we differentiate between the structure and drivers of the geographically rare and common alien and native understorey plants. Distinct differences were identified between the alien and native species; namely that natives were driven by distance, while aliens were driven by environmental filtering and soil pH constrained both common natives and rare and common aliens.

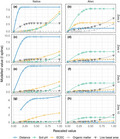

## 1 INTRODUCTION

Species invasions are a leading global threat to biodiversity and new introductions show no signs of slowing (Seebens et al., 2017). Vascular plants represent almost half of all known introductions, with Oceania (including Australia) among the most impacted regions on Earth (Seebens et al., 2017; van Kleunen et al., 2015). However, introductions have highly variable effects on plant community structure and diversity (Gaertner et al., 2009; Jackson & Sax, 2010; Sax & Gaines, 2008; Simberloff, 2001), and the role of different environmental factors in the outcome of multispecies invasion of communities remains unclear (Brummer et al., 2016). For any factor influencing plant performance, alien and native species might potentially respond in the same or opposite direction (Brummer et al., 2016). Alternatively, they might depend on entirely different factors, or the response could depend on the species relative abundance (Brummer et al., 2016; Powell et al., 2011). Each situation might inform different management interventions to protect native species. If, for example, certain conditions are associated with more problematic alien species performance, management efforts might prioritize areas where these conditions are found, but alien presence is currently low (Catford et al., 2012). Multispecies introductions provide an opportunity to understand what determines the commonness and rarity of naturalized non‐native (alien) species and whether this differs from the determinants for native species within a single study system (Bernard‐Verdier & Hulme, 2015; Brummer et al., 2016; Latombe et al., 2018).

Once established in a landscape, alien species become part of the spatial and temporal dynamics of local biodiversity (Bernard‐Verdier & Hulme, 2015). However, whether this results in an impact on the native community depends on the ecological pattern considered (Pyšek et al., 2012), and inference based on individual measures of community structure can be misleading. For example, a focus on species richness can result in misrepresentation of the impact of aliens on the recipient system if both the loss of natives and their replacement by alien species are not considered (Hillebrand et al., 2018). This highlights the value of considering the native and alien species components independently (Bernard‐Verdier & Hulme, 2015; Brummer et al., 2016) and the need to consider multiple measures of community structure (McGill et al., 2015). Complementing more traditional metrics used to describe community structure (e.g., species richness, abundance, composition), methods based on zeta diversity (Hui & McGeoch, 2014) have shown that the determinants of multispecies turnover can differ between narrowly and more widespread species (Latombe, Richardson, et al., 2018; McGeoch & Latombe, 2016; McGeoch et al., 2019). Together, these methods support tests of the relative influence of spatial and abiotic drivers of diversity and composition for rare vs common and alien vs native species.

Patterns of species diversity and composition reflect stochastic and deterministic sorting of species along abiotic gradients, limitations to their dispersal, and the outcome of local‐scale interactions among species (Leibold et al., 2004; Logue et al., 2011; Mouquet & Loreau, 2002). If alien and native plants (or rare or common species within either component) differ in their abiotic tolerances, dispersal abilities, or life history strategies, this should be evident in the predictors that best explain their respective patterns of diversity and compositional turnover. Rare species are often considered specialists with narrower tolerance of abiotic conditions, whereas common species are considered generalists with broader niches (Brown, 1984; Okimura & Mori, 2018). Assuming all species can reach locations offering optimal abiotic conditions, rare species turnover should then be more sensitive to abiotic conditions than more common species, regardless of whether their origin is native or alien. Conversely, under more extreme abiotic conditions, alien species might be more disadvantaged either because of physiological limits to establishment, lower propagule pressure, or relatively high competition from native species (Alpert et al., 2000; Zefferman et al., 2015). In this case, alien species might be more subject to a filtering effect of abiotic conditions than native species, which could impact diversity and turnover.

Alien species are often associated with more efficient dispersal, but evidence for this is inconsistent and high rates of spread can also reflect success through other stages of establishment (e.g., germination, seedling survival; Flores‐Moreno et al., 2013). If alien species were able to spread more effectively than natives, this should homogenize their composition across sites (Mouquet & Loreau, 2003) and result in lower compositional turnover particularly among the most common and widespread alien species. Any difference in the life history strategies between alien and native species (e.g., *r* vs *K* selection) could also influence their establishment success and therefore compositional turnover, but evidence for differences between native and non‐native species in this regard is inconsistent (Pyšek & Richardson, 2007).

The presence of multispecies introductions within a network of conservation reserves in south‐eastern Australia presents an ideal study system to test whether the correlates of diversity and turnover differ between alien and native species and whether these vary between rarer or more common species in either component. Here, we test for such effects by separately analyzing the richness, relative frequency, and turnover of the native and alien species components in the understory plant community of eucalypt‐dominated dry forest. As native species should include more narrowly distributed (specialized) and fewer widespread (generalist) species than the alien component, we expected higher turnover among native species and a more pronounced distance decay in response to spatial autocorrelation in abiotic conditions (Nekola & White, 1999). In contrast, we expected alien species distributions would be more sensitive to environmental filtering from abiotic conditions, where more extreme local conditions (e.g., low pH soils) would impose greater influence on their richness, relative frequency, and turnover.

## 2 METHODS

### 2.1 Study region and species

**FIGURE A1:**
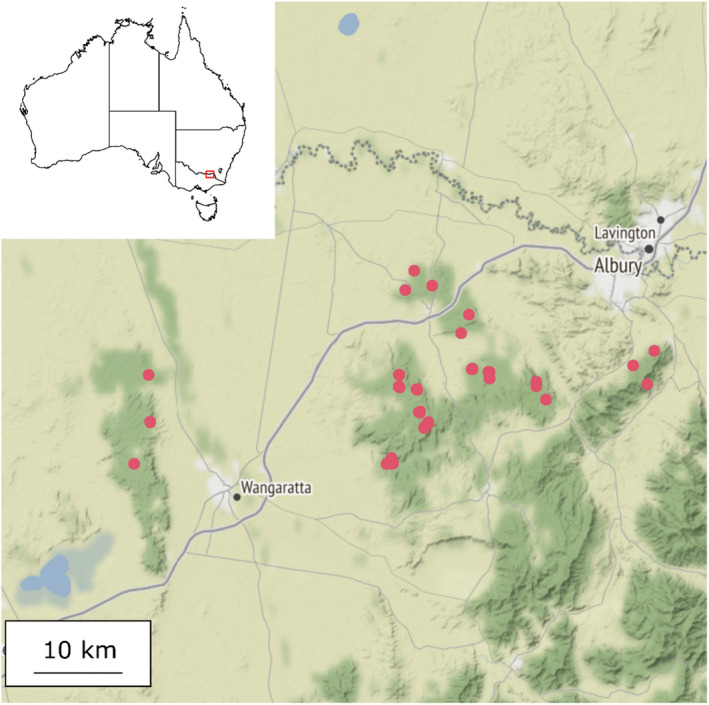
Location of the study area and 50 plots examined within the project

The understory plant community of eucalypt‐dominated dry forest was sampled across four conservation reserves (total study extent ~1030 km^2^) located along the inland slopes of the Great Dividing Range in northern Victoria, Australia (Figure A1, Table A1, Appendix A). The region has a warm temperate climate, with mean annual daily maximum temperature ~22°C. Mean annual rainfall is around 600 mm. Rainfall in the winter‐spring months tends to be greater and more predictable. Soils in the region are predominantly texture contrast (e.g., chromosols and sodosols) of moderate‐strong acidity (soil pH range: 4.5–6.9; Table A2) and low‐moderate chemical fertility (McGrannachan & McGeoch, 2019).

**FIGURE A2:**
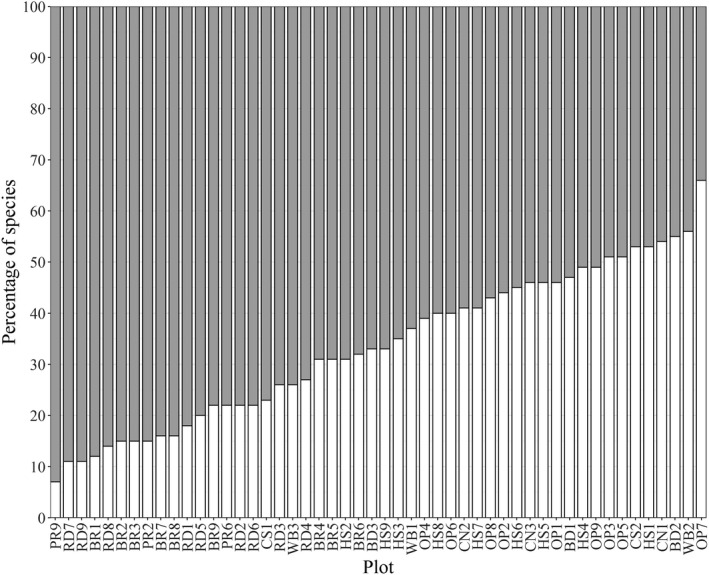
Ranked level of invasion per plot shown by percent richness (where richness refers to the incidence of a species within a plot). Alien species are shown in white and native species in grey

**FIGURE A3:**
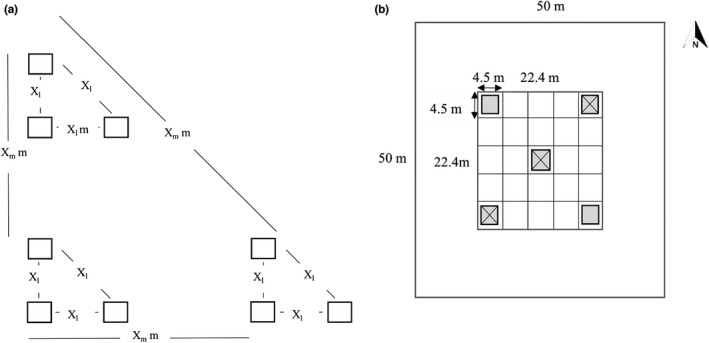
Hierarchical array sampling design implemented across the four conservation reserves (Chiltern Box‐Ironbark National Park, Mt Pilot Park, Baranduda Regional Park and Warby Ovens National Park; Figure A1). (a). Each site comprised three plots (shown by squares). Inter‐plot distances (X_l_ m) varied among sites, but ranged from 30–100 m. The distance between sites (X_m_ m) also varied but ranged from 0.4–1 km. (b) Detail showing the design of the forest structure (50 × 50 m) and understory (22.4 × 22.4 m) plots. The understorey plot was divided into a grid of 25 sub‐plots (4.5 × 4.5 m). Soil samples were taken at each of the corner and centre sub‐plots (shown in grey shading) and leaf area index photographs were taken in sub‐plots 5, 13 and 25 (marked with crosses)

Understory plants were sampled along a gradient of alien plant introductions, inferred from the proportion of alien species present in the understory (6–65% alien plant species richness; Figure A2, Appendix A). Vertical structure of the understory is generally limited to a ground layer comprising ferns, forbs, and graminoids, with only sparse presence of shrubs (fewer than 5% of subplots) rather than a defined layer. There were no entirely uninvaded understory areas in the region, but canopies are predominantly native, with few alien species or individuals (McGrannachan & McGeoch, 2019). We used a nested hierarchical sampling design; where at the highest level, we established sites comprising three 2500 m^2^ (50 × 50 m) forest structural plots located between 30 and 100 m apart at the nearest point (Figure A3). Centered within each structural plot was a 500 m^2^ understory plot, which formed our unit of analysis. Each plot was divided into a sampling grid of 25 contiguous square quadrats each of 20 m^2^ (i.e., 4.47 × 4.47 m; Figure A3). Plots were positioned within homogeneous stands of forest or woodland vegetation typical of the region, situated at least 200 m from park boundaries and at least 100 m from roads, waterways, and edge habitats. Sites range from 170 to 572 m above sea level in elevation and were selected to ensure elevational differences across the three nested structural plots were less than ~30 m. Further technical details on sampling design can be found in McGrannachan and McGeoch (2019) noting the current study extends the approach over a wider extent.

Field surveys were conducted by experienced botanists during the late spring and early summer months (Sep–Dec) from 2013 to 2017 (Table A3). Species identification followed the nomenclature of Walsh and Entwistle (1992–1996) and data on species origin (native or alien) were obtained from VicFlora (2019). Where plant identification was ambiguous, specimens were classified using identification keys or were returned to the laboratory for further confirmation (Costermans, 2009). The presence–absence of each species within each of the 25 subplots in the 500 m^2^ understory plot (Figure A3b) was recorded and from this we calculated a frequency of occurrence for every species in the plot, comprising a value between 1 and 25 representing the integer number of subplots in which it was present (Kent, 2011). In total, the data comprised 37,553 observations of 251 understory species, predominantly graminoids and herbs.

### 2.2 Environmental data

To quantify variation in the broad environment at the level of plots, we recorded the elevation using a handheld GPS and measured the slope and aspect of each plot using a compass clinometer. We also quantified (i) overstory structure in the larger forest plot at each site using total live basal area (m^2^ ha^−1^) of all individuals with diameter at breast height exceeding 100 mm and (ii) leaf‐area index for the 500 m^2^ understory plots (Figure A3). To characterize the plot‐level soil environment, we analyzed soil chemical, micro‐ and macro‐nutrients from pooled subsamples collected from the four corner subplots and the center subplot of each plot (Figure A1, Table A1, Appendix A). In total, we had 19 environmental variables – four relating to the overstory structure and physiographic setting and 15 quantifying soil chemical and nutrient values. We selected a subset of these as predictors as described below.

### 2.3 Statistical analysis

**FIGURE A4:**
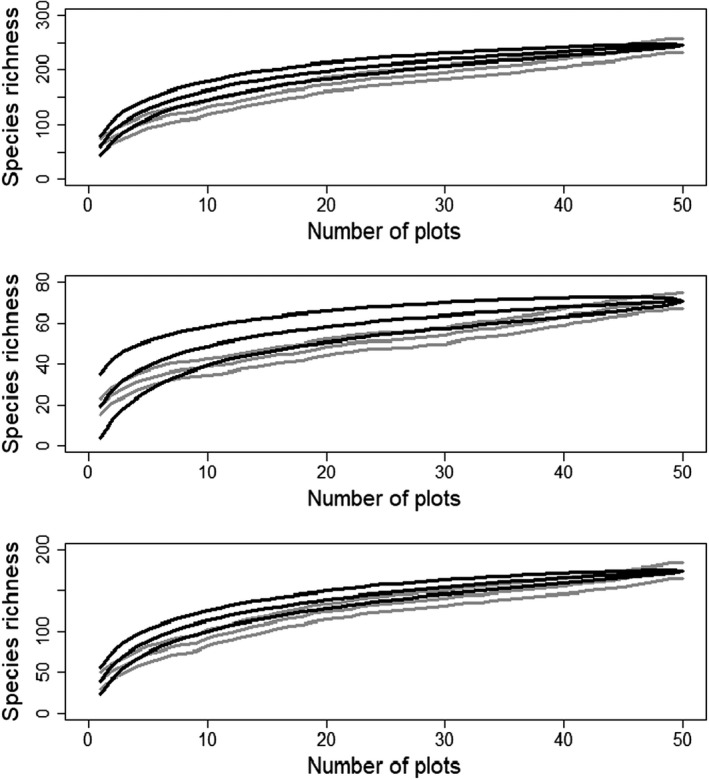
Species accumulation curves (rarefaction curve with 95% confidence interval) for (a) the whole community, (b) the alien community and (c) the native community. Where the standard rarefaction curve is shown in black and spatially constrained curve in grey

Prior to analysis, we build plot‐scale rarefaction curves from presence–absence transformed data for all species combined and separately for the alien and native components, comparing spatially constrained (i.e., combining plots in order of proximity) and fully randomized rarefaction curves to assess the effects of spatial aggregation. Rarefaction curves and 95% confidence intervals were created using 1000 permutations (Figure A4). Sampling adequacy was evaluated using the Chao2 non‐parametric species richness estimator (Chao, 1987), with native and alien species richness each estimated to be ~84% complete.

**FIGURE A5:**
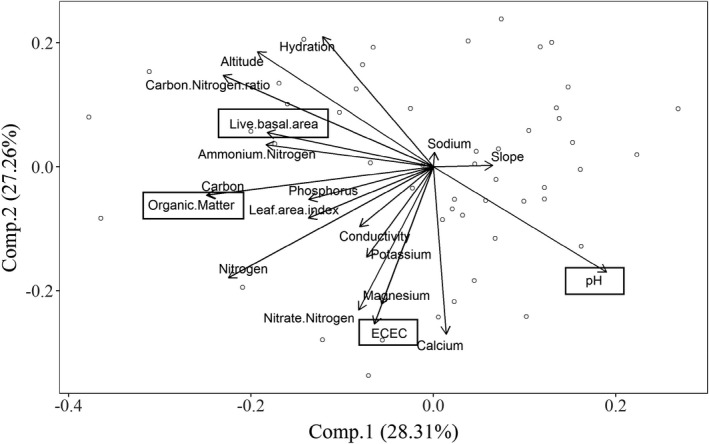
Principal component analysis (PCA) of the environmental variables used in selecting predictors for modelling. The names of variables selected for use in the explanatory models are indicated by an enclosing box. The first four axes of the PCA explained 74% of the variance, but over 55% was contained within the first two principal components (proportion of variance explained: Comp 1 = 28%, Comp 2 = 27%, Comp 3 = 10% and Comp 4 = 9%

**FIGURE A6:**
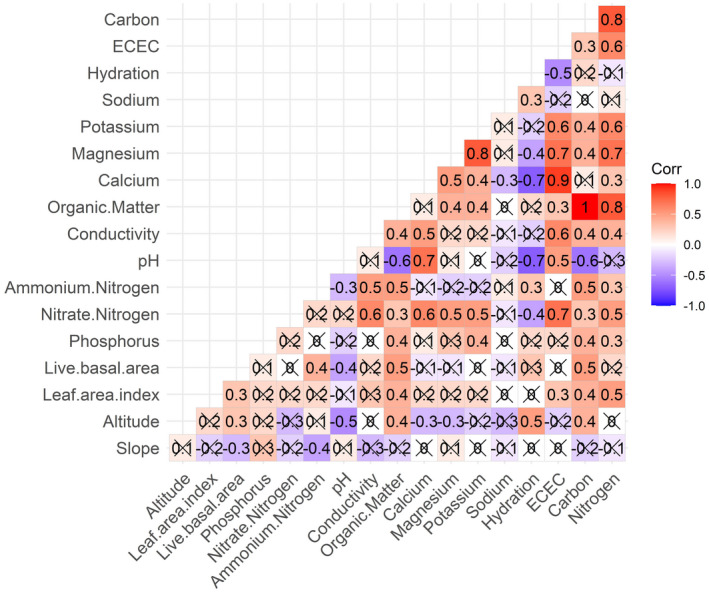
Pearson's product moment correlation among the full set of environmental variables. Selected environmental predictors were tested to ensure any correlation was below 0.6 before adopting. Crosses represent non‐significant correlations (*p* > .05)

For both alien and native species components of the understory, we repeated the same three sets of analyses of diversity and turnover. We modeled (i) species richness and prevalence (as frequency in subplots) using regression models; (ii) analyzed turnover using zeta diversity decline (McGeoch et al., 2019), and (iii) used dissimilarity modeling (Latombe et al., 2017) to differentiate the role of spatial and environmental factors. To maintain adequate degrees of freedom to estimate the regression models, we decided a priori to limit the number of predictors to 4. To select these from among the 19 available environmental variables, we used principal component analysis. We identified relatively un‐correlated variables based on the magnitude and relative position of their loadings in a plot of the first two principal components (accounting for 55% of total environmental variation; Figure A5). The selected predictors were soil organic matter, live basal area, effective cation exchange capacity, and soil pH (see Figure A6 for the Pearson product moment correlation between all environmental variables).

To model species richness of alien and native plant species at the understory‐plot level, we used a generalized linear model. We calculated species richness of each plot by converting the frequency data to presence–absence and modeled this as a function of environmental predictors. We used a negative binomial error structure due to overdispersion in the species richness (count) data. All models contained our set of four environmental variables in a linear combination (i.e., no interactions) and inference was based on the full model. This a priori approach to selection of predictors avoids the issue of artificially inflating Type 1 error probabilities (Head et al., 2015) and acknowledges uncertainty in the ability of regression‐type approaches to identify causal mechanisms. However, interpretation requires recognition that highly correlated predictors could have resulted in similar model fits. For example, effective cation exchange capacity had notable (>|0.5|) correlations with macro and micronutrient concentrations (e.g., nitrogen, potassium, calcium, and magnesium), pH covaried negatively with elevation, while organic matter and live basal area were associated with total nitrogen and the carbon‐to‐nitrogen (C:N) ratio (Figure A6). We confirmed no serious spatial autocorrelation in model residuals using Mantel tests of residual and spatial distance matrices (all *p* > .1).

To determine whether the relative frequency of native and alien species at the plot scale depended on environmental conditions, we used multivariate generalized linear models (manyGLM) (Wang et al., 2012). This approach fits a separate model for each species and calculates a multivariate test statistic by resampling from the individual results (Warton et al., 2012). We used the plot‐scale relative frequency data as our response variable and built models with the same structure as for species richness. All regression modeling was done using R (R Core Team, 2019) with packages “vegan” (Oksanen et al., 2020), “MASS” (Venables & Ripley, 2002), and “mvabund” (Wang et al., 2012).

To test compositional turnover in the alien and native components, and for groups of species with different occupancy within them, we used zeta diversity – the number of species shared among a group of samples (Hui & McGeoch, 2014). Zeta diversity of order *i* (denoted “ζ_i_”) quantifies the number (or proportion) of species shared across *i* samples. With increasing order, zeta diversity reflects turnover in only those species with higher occupancies, while at low orders all species contribute to the observed value. For example, the mean number of species found in one site (zeta diversity of order 1) is identical to alpha diversity. The mean number of species shared by two sites, ζ_2,_ is a measure of pairwise similarity, with a clear (though complementary) relationship with metrics of pairwise beta diversity (e.g., Jaccard dissimilarity). For orders of zeta greater than 2, there are no analogous metrics of turnover. Therefore, an advantage of the zeta diversity partition is the ability to use a single metric to explore the influence of spatial and environmental factors on the turnover of narrowly vs widely distributed species (McGeoch et al., 2019).

We examined the drivers of turnover in alien and native understory plants in two ways (i) by quantifying the change in shared species with increasing numbers of sites (zeta decline) and (ii) by separately examining the effects of spatial and environmental factors for rare to common species using generalized dissimilarity modeling (Ferrier et al., 2007) for multiple sites [multisite generalized dissimilarity modeling (MS‐GDM); Latombe et al., 2017]. Zeta decline is the change in the average number of shared species across the landscape as additional sites are considered.

Zeta decline provides insights on drivers of spatial turnover through comparison of different normalization and subsampling schemes. We compared raw zeta decline (the arithmetic mean of the number of shared species) with the Simpson‐equivalent normalization, where the number of shared species is divided by the minimum richness of the sites being combined (McGeoch et al., 2019). Comparing raw zeta decline with the Simpson‐equivalent provides analogous insights to comparing the total and turnover component of beta diversity (Baselga, 2010). Spatial dependence in turnover can be examined by comparing zeta decline when subsampling sites at random (the “ALL” subsampling scheme) with that calculated from constraining all combined sites to nearest neighbors (the “NN” subsampling scheme). Selection of non‐directional nearest neighbors to quantify compositional turnover accounts for distance decay of compositional similarity (McGeoch et al., 2019) and thus comparison of the “ALL” and “NN” results reveals the importance of spatial proximity for compositional similarity. For zeta orders where the two curves overlap, the probability of sharing species of that order does not depend on the distance between the samples. Zeta decline for orders 2–50 was quantified using Monte Carlo sampling with 10,000 replicates (Latombe et al., 2018a).

To understand the role of both spatial distance and environmental gradients for turnover, we used MS‐GDM (Latombe et al., 2017). Unlike univariate linear regression, MS‐GDM models the difference in shared species for a group of sites as a function of their spatial and environmental distance, providing an indication of the relative importance of both factors in turnover for different orders of zeta. As the predictive power of MS‐GDMs tends to decline with increasing order of zeta, we limited modeling to a maximum of zeta 10 (Latombe et al., 2017). A separate analysis for all orders (2–10) was done for both the native and alien components using the four environmental variables and distance between plots. In the results, we illustrate the observed trends using zeta orders 2, 4, 5, and 10. The same environmental predictors were used as for regression modeling and models were fit using I‐spline regression (Latombe, McGeoch, et al., 2018) from 10,000 randomly selected sets of plots. Models are interpreted based on the relative magnitude and shape of the I‐splines for turnover in species composition (Ferrier et al., 2007; Latombe et al., 2017) and the variation explained partitioned into independent fractions associated with distance and abiotic factors and their combined effect (Latombe, McGeoch, et al., 2018). All compositional turnover modeling used custom R package ‘zetadiv’ (Latombe, McGeoch, et al., 2018).

## 3 RESULTS

### 3.1 Diversity of the alien and native plant components

**FIGURE 1:**
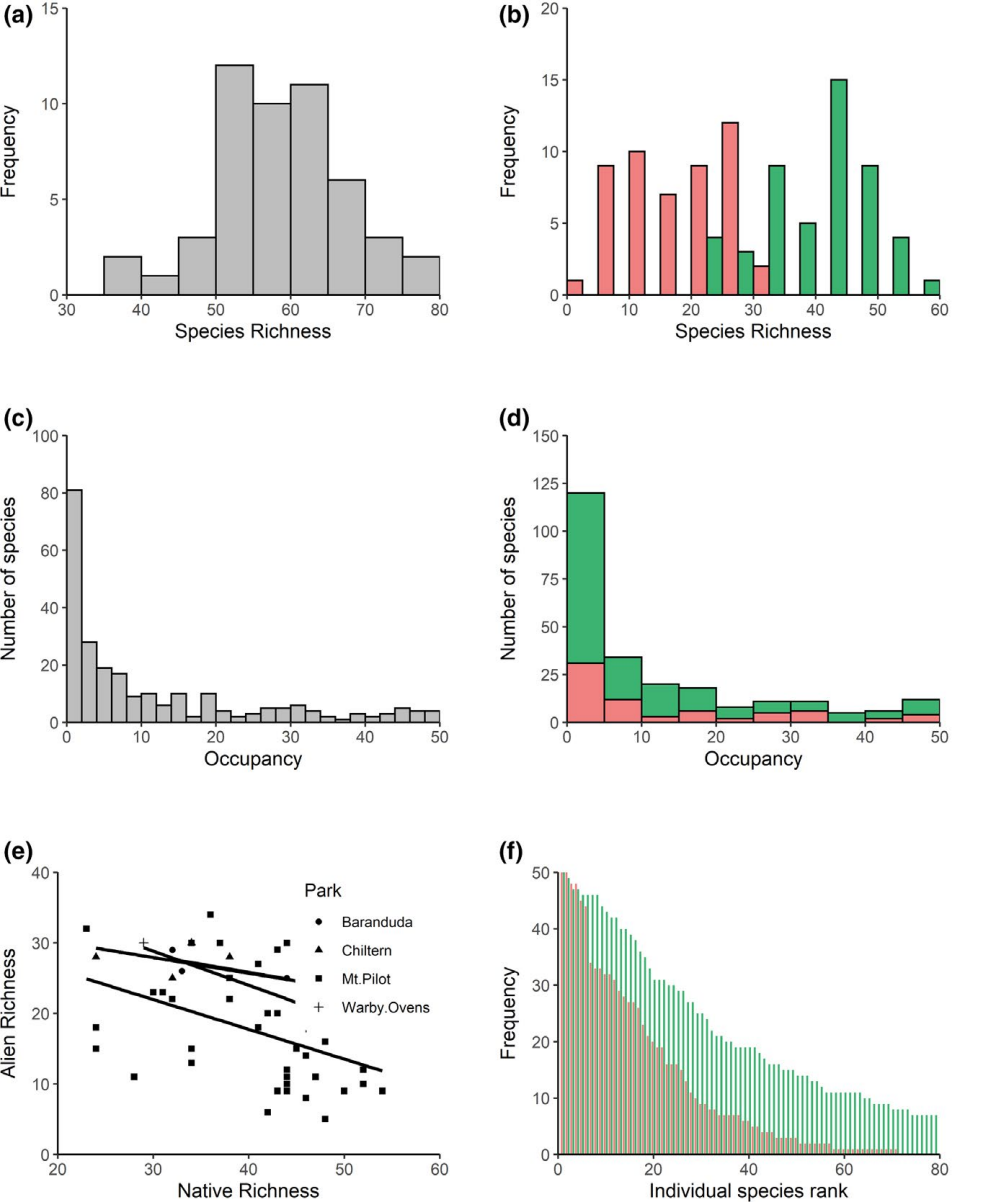
Patterns of richness and plot‐level occupancy in the understory community. Top row shows species richness frequency distributions of (a) the entire plant community and (b) the alien (red) and native (green) components (*n* = 50). Middle row shows species occupancy distributions across (c) the entire plant community and (d) of the proportion of alien (red) and native (green) components. Bottom row shows (e) Relationships between alien and native species richness at each park (adjusted *R* ^2^ = 18.6% across all parks) and (f) Rank frequency distributions (within‐plot occupancy) of all alien species and the top 80 most common native species

**FIGURE 2:**
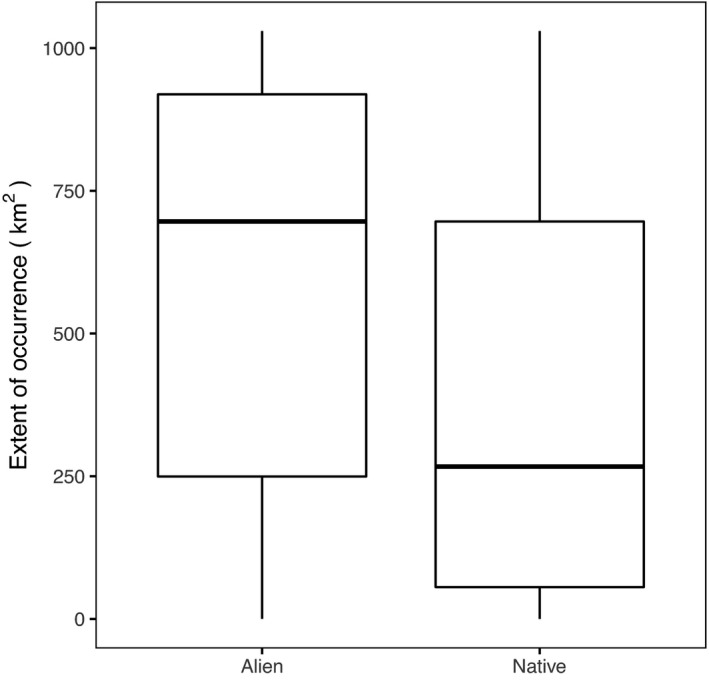
Extent of occurrence of all alien and native species observed in at least three plots (50 alien and 117 native species). Extent of occurrence was estimated as the area of the minimum bounding polygon for all plots where the species was present. Total study extent calculated from this method was 1029 km^2^

Of the 251 species identified in the understory plant community, 178 (71%) were native and 73 (29%) were alien to the region (Table B1, Appendix B). Alien species were mainly (79%) short‐lived annuals, while native species were predominantly (82%) perennial in both common and rare species (Tables B2 and B3, Appendix B). Native species richness was higher on average than alien species richness (mean difference = 20.8 species, *t* = 10.2, *df* = 49, *p* < .001; Figure 1, Table B1), but there was a negative association between them (regression slope −0.48, *t* = −3.6, *p* < .001, *R* ^2^ = 0.20; Figure 1a). In contrast, within‐plot frequency for alien and native species was positively correlated (slope = 0.27, *t* = 3.08, *p* = .004; *R* ^2^ = 0.33). Although the occupancy for native species appears more skewed toward lower values (Figure 1b,f), there was no evidence they followed a different distribution (Kolmogorov–Smirnov test, *D* = 0.08, *p* = .8). However, alien species tended to have a greater extent of occurrence (EOO) within the study area than natives (median EOO: aliens = 696 km^2^, natives = 267 km^2^; KW χ^2^ = 9.2, *p* < .001; Figure 2).

Species richness of alien plants was better explained by environmental conditions than natives (pseudo *R* ^2^ = 0.53 vs. 0.29, Table 1), with species richness increasing at higher pH and decreasing with higher live basal area (*p* < .05; Table 1). Post hoc tests show that even the most tolerant alien species found at low pH attained significantly lower within‐plot relative frequencies where soil pH was below 5.5 (0.29 vs 0.54; KW test; *p* < .001). None of the environmental predictors individually affected native plant species richness (all *p* > .1; Table 1), despite collectively explaining nearly one‐third of variation (regression pseudo *R* ^2^ = 0.29). However, adopting a definition of rare native species as those found in fewer than 20% of plots, both the richness (8 vs 5 species, *p* < .001) and relative frequency (0.018 vs 0.010, *p* = .005) of rare native species was higher when soil pH was below 5.5. This post hoc result is consistent with an environmentally constrained impact of alien species on rare natives that warrants appropriate testing.

**TABLE 1:** Regression model results for species richness of alien and native species as a function of environmental predictors

Model	Predictor	Est	SE	*z*	*p*
Alien species *R* ^2^ 0.53	Organic matter	−0.04	0.10	−0.45	.66
	**Live basal area**	**−0.12**	**0.05**	**−2.33**	.**02**
	Effective cation exchange capacity	0.10	0.08	1.23	.22
	**pH**	**0.19**	**0.07**	**2.02**	.**04**
Native species *R* ^2^ 0.29	Organic matter	0.01	0.05	0.11	.92
	Live basal area	0.05	0.03	1.57	.12
	Effective cation exchange capacity	−0.05	0.05	−0.97	.33
	pH	−0.06	0.06	−1.11	.27

Note

Models were fit using a negative binomial error. Coefficients significantly different from zero (*p* < .05) are shown in bold. See Table A2 for a summary of the range of values for the environmental predictors.

**FIGURE 3:**
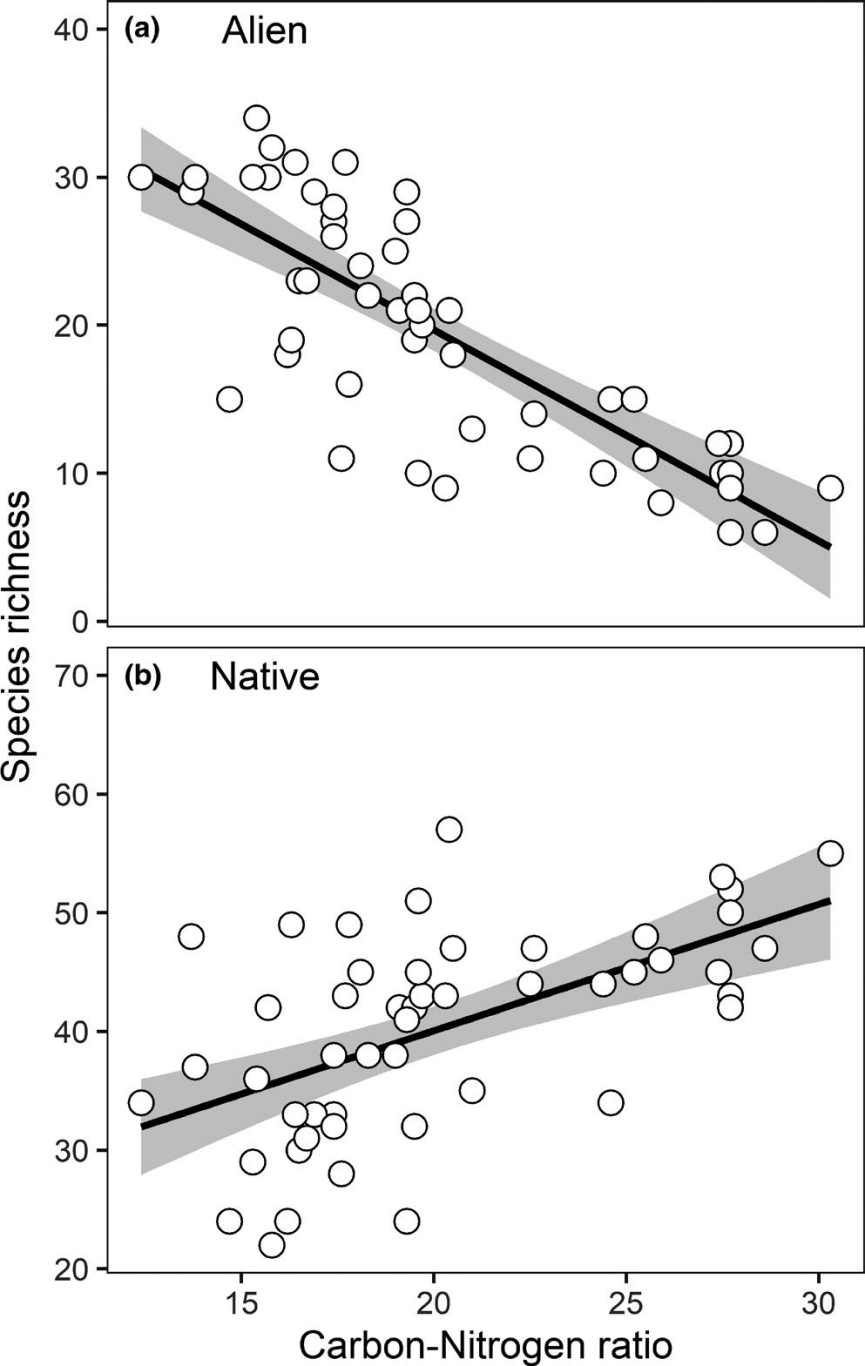
Sensitivity of (a) alien and (b) native species richness to plot‐scale soil carbon‐nitrogen ratio. Note alien species richness was constrained at values exceeding ~20 in the C:N ratio. Lines are ordinary least squares regressions and shading shows one standard deviation in the slope

Within‐plot frequency followed broadly similar trends, with alien plants tending to be of lower frequency in plots with higher live basal area (median coefficient value across all species = −0.31, *p* = .02, Table 2) but greater frequency in locations with higher effective cation exchange capacity (median coefficient = 0.27, *p* = .01, Table 2). Live basal area was strongly correlated with the C:N ratio and as nitrogen mineralization decreases with increasing C:N ratio, lower nitrogen availability (as opposed to live basal area per se) could account for the lower number of alien species at sites where C:N ratios exceeded ~20 (Figure 3). Evidence of any environmental effects on native species within‐plot frequency was equivocal, with only marginal evidence (.05 < *p* < .1; Table 2) for positive effects of effective cation exchange capacity and live basal area and a negative effect of organic matter (median coefficient values: 0.01, 0.03, and −0.04, respectively, Table 2).

**TABLE 2:** Multivariate regression model of alien and native plant frequency as a function of environmental predictors

Model	Predictor	LR	*p*
Alien species	Organic matter	98	.16
	**Live basal area**	**145**	.**02**
	**Effective cation exchange capacity**	**148**	.**01**
	Soil pH	105	.08
Native species	Organic matter	278	.08
	Live basal area	312	.07
	Effective cation exchange capacity	287	.06
	Soil pH	228	.17

Note

Models assumed a negative binomial error structure. Coefficients significantly different from zero (*p* < .05) are shown in bold. See Table A2 for a summary of the range of values for the environmental predictors.

Abbreviation: LR, likelihood ratio.

### 3.2 Compositional turnover

**FIGURE 4:**
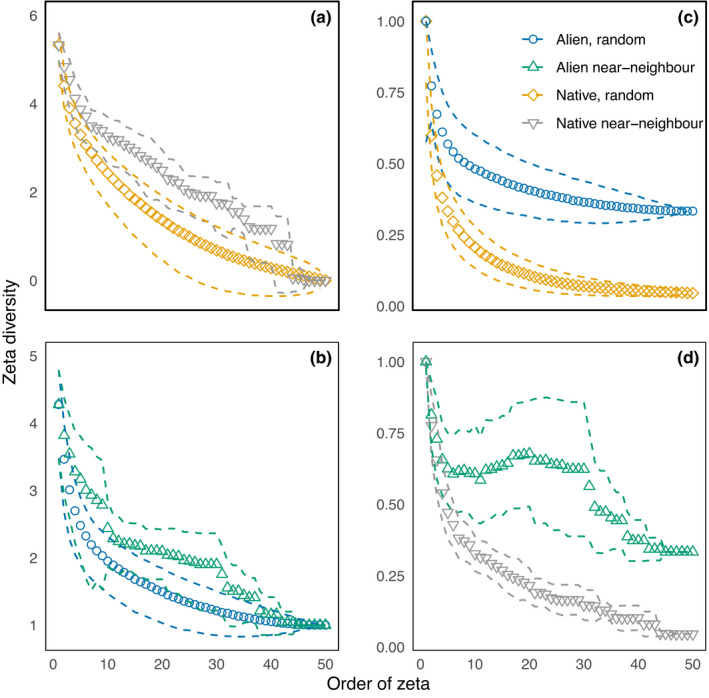
The decline in compositional similarity across sites (zeta decline) for the (a) native component (green) and (b) alien component (red) of the plant community – using raw zeta values (a, b, note log‐scale) and Simpson‐normalized values (c, d). The decline in shared species was calculated for random combinations (ALL; circles), which can be directly compared with spatially explicit subsampling (NN; crosses), which preferentially selects the nearest neighbors for comparison. Differences between the sampling variability thus provide an indication of the importance of dispersal limitation in structuring turnover

Multisite compositional turnover declined according to a power law, rather than an exponential form for both native and alien species but was higher in the former (exponent [95% CI] for power law model: natives = −0.84 [−0.87, −0.82]; aliens = −0.26 [−0.31, −0.19]; Figure 4a,b). Only one native species was shared across all plots, despite having more than twice the total landscape richness of the alien component, which shared two species (Figure 4). Both alien and native components showed a rapid decline in the average number of species shared over the first three orders, after which the rate of decline diverged, retaining a much higher value in alien species (Figure 4a,b). This is most clearly shown by the Simpson‐normalized zeta decline, where shared alien species reached a minimum of around 0.4 of minimum plot richness shared across all sites (ζ_50_), while for native species the equivalent value was 0.04 (Figure 4c). Spatial proximity of sites was more influential for turnover in the native component, with the nearest‐neighbor curve (Figure 4a, dashed lines) consistently above the random curve and with little overlap in confidence intervals to an order of around 30.

**FIGURE 5:**
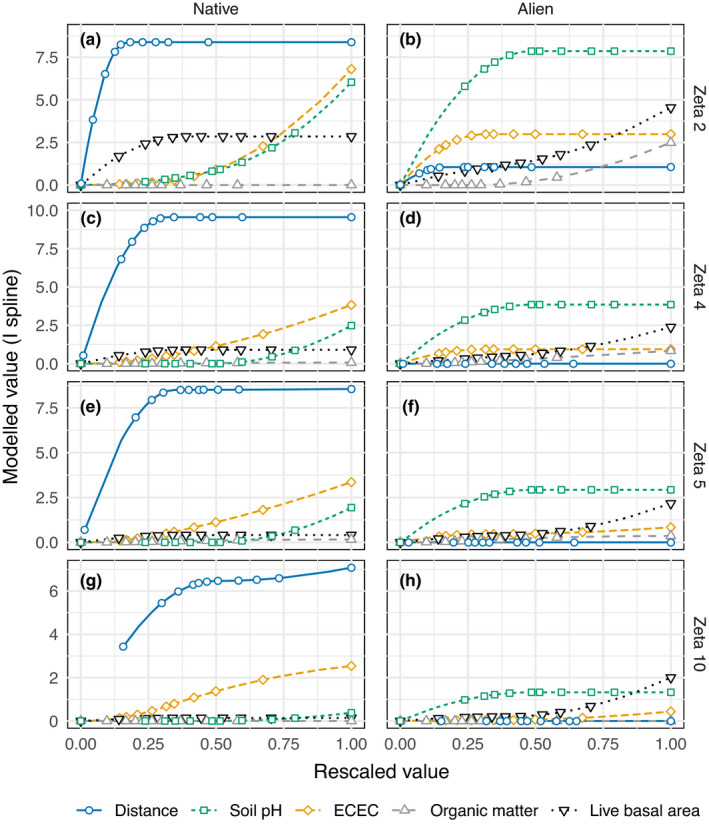
The influence of distance and local abiotic conditions (ECEC = effective cation exchange capacity) on compositional turnover in species of increasing occupancy for the two vegetation components (left column, panels a, c, e, g = native; right column, panels b, d, f, h = alien species). Each row shows the influence of the predictors on turnover of species found in a different number of sites, increasing from top (Zeta 2) to bottom (Zeta 10). I‐splines show how these relationships change across the rescaled ranges of the environmental variables and distance, using I‐splines from MS‐GDM. Each explanatory variable is transformed using I‐splines scaled from low (right) to high (left) values of the variable. A steep slope between the original (rescaled range; x‐axis) and transformed (I‐splines; y‐axis) variable indicates high rate of turnover, and the larger the value (height) of the variables I‐spline on the y‐axis, the larger its explanatory power. Points show the location of the sampled sites for deciles of the raw values of the predictor (see Figure B3 for plots of each predictor using the original scale and Table A2. for explained variation). Values were calculated from raw zeta values for 10,000 combinations of sites

In contrast, confidence intervals for the alien component overlapped over most of the range of orders considered (Figure 4b), although this was at least partly because of greater variability in the role of spatial proximity for alien species turnover (e.g., comparing the width of the confidence intervals for alien and native species in Figure 4a,b). A larger proportion of alien species were widespread compared to the native component (Figure 4c,d; the difference in zeta values at highest orders; see also Figures 2 and 5). The decline of the Simpson‐normalized zeta diversity for the alien component did not follow a typical monotonic decline when using the nearest‐neighbor subsampling scheme, with compositional similarity increasing across some orders, showing that common alien species seem to co‐occur at the same spatially clustered plots (between ~zeta orders 5–30; Figure 4d). The form of zeta decline for both alien and native components was best described by a power law (in both cases ΔAIC > 10 below the exponential model).

### 3.3 Environmental and spatial effects on turnover

The variables that explained turnover in species composition differed between the native and alien components and, to a lesser extent, between occupancy classes within them (Figure 5). Distance accounted for up to half of the explained variation for native species (range 0.04–0.12, Table B4), but this decreased among more common species (i.e., zeta 10; Figures 5 and B3, Table B4). Environmental factors explained a similar fraction of variation to that explained by distance for natives (range 0.03–0.12, Table B4) but again explained little variation in common species. Soil pH, live basal area, and effective cation exchange capacity were each important for rare native species turnover, but only the latter influenced common species (Figure 5. left column, Figure B3, Table B4). Alien species turnover was essentially the opposite of natives, being driven largely by environmental variation and with distance playing almost no role (Figures 5 and B3, Table B4). Soil pH and live basal area were important for turnover of all alien species (from rare to common), but effective cation exchange capacity was of moderate importance only for low occupancy alien species (i.e., zeta 2). Unlike native species, explained variation in alien species was essentially constant across orders of zeta (each explaining ~30% of variation; Table B4). Changes in the influence of between‐plot distance for the native component were most pronounced over small distances (<10 km), while for the alien component, turnover was sensitive to small changes in environmental conditions, particularly soil pH < 5.5 (Figure B3, Appendix B).

## 4 DISCUSSION

Understanding what influences the contribution of native and alien species to community structure can help better understand the impacts of species introductions, direct future research, and inform management interventions to maintain biodiversity (Bernard‐Verdier & Hulme, 2015; Brummer et al., 2016). We illustrate the information value of separately considering the drivers of diversity and relative commonness of each component separately (Latombe, Richardson, et al., 2018; McGeoch et al., 2019) and show that whereas the composition of the native community was predominantly explained by distance, alien species composition was best explained by environmental variation. Coexistence of alien and native species at landscape scales appears to be mediated by environmental conditions, while at local (within plot) scales, trade‐offs in life history strategy (e.g., annual vs. perennial growth habit) appear most important.

### 4.1 Environmental conditions explained diversity and turnover in alien but not native species

Alien species richness, relative frequency, and turnover were largely explained by the harshness of abiotic conditions, consistent with the lower invasion of such habitats (Zefferman et al., 2015) and environmental filtering of alien species (Weiher & Keddy, 1995). Soil pH was particularly influential and only the most common and widespread alien species (Figure B2) were present in low pH plots. As is typical in Australia, soils in this landscape are acidic and most plots fell outside the optimal pH range of 6–8 (Läuchli & Grattan, 2017). This environmental filtering effect appears to constrain the distribution of alien species and strongly influenced their diversity (Ulrich et al., 2017). Similarly, alien plants appeared more prevalent at sites with higher effective cation exchange capacity and lower live basal area, both potentially indicating relatively high nutrient availability. However, increasing live basal area would also reduce light availability and its negative influence on the alien understory could reflect shade intolerance (Bernard‐Verdier & Hulme, 2015; Keeley et al., 2005).

### 4.2 Correlates of turnover differ between common and rare, alien, and native species

Patterns of compositional turnover also differed between native and alien species and among rare and common occupancy species within them. As expected, the native component exhibited greater turnover due to more narrowly distributed species. As this appears unrelated to the measured abiotic conditions, the effect of historical contingency (e.g., the order of species arrival during assembly; Fukami, 2015) or unmeasured climatic or environmental variables (e.g., water availability; Flanagan et al., 2015) could influence vegetation structure and result in a predominantly neutral process of distance decay (Hubbell, 2001) within more physiographically distinct settings.

**FIGURE B1:**
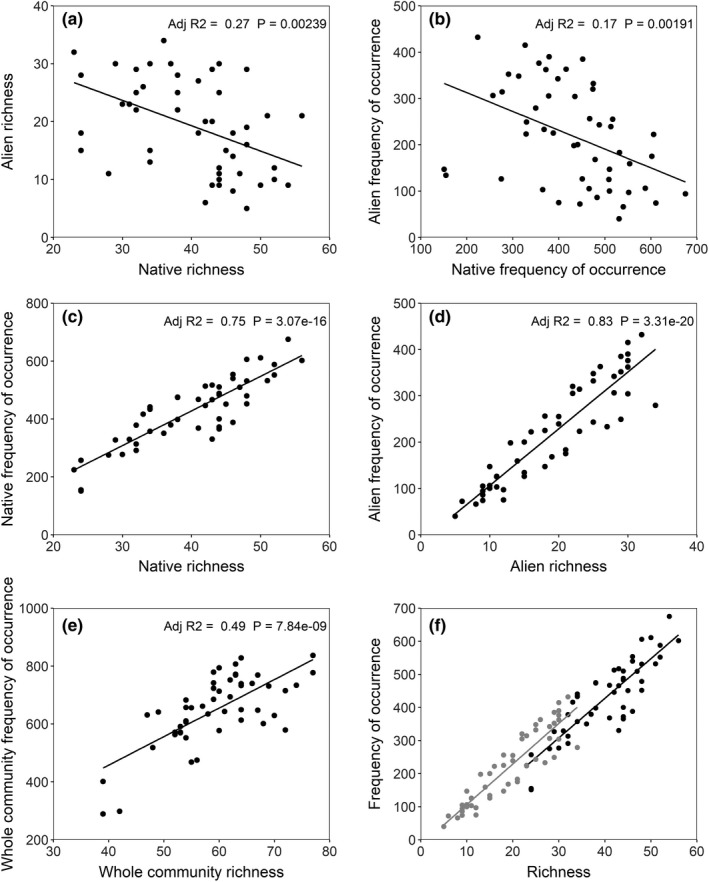
Relationships between (a) alien and native richness, (b) alien and native frequency of occurrence, (c) native richness and frequency of occurrence, (d) alien richness and frequency of occurrence, (e) whole community richness and frequency of occurrence and (f) alien (grey) and native (black) richness and frequency of occurrence

**FIGURE B2:**
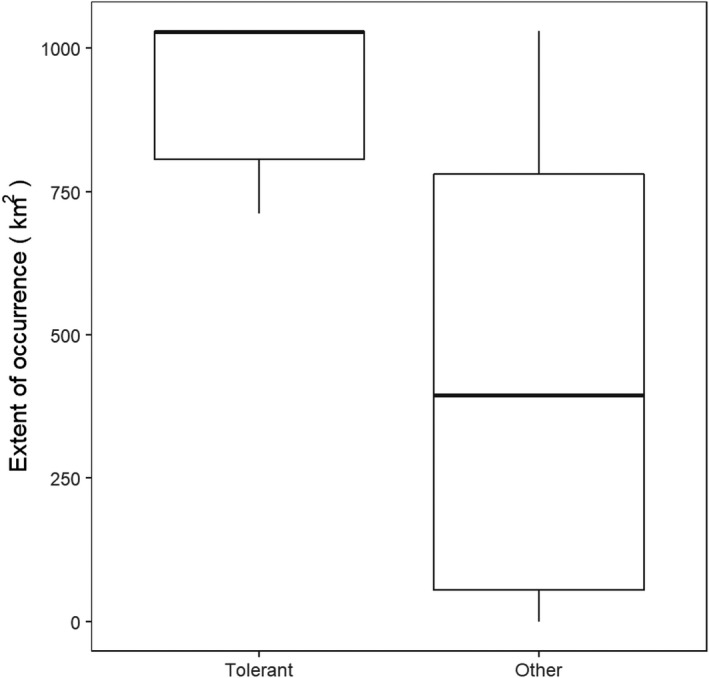
Extent of occurrence of alien species comparing Tolerant and Other species. Classification of species as ‘Tolerant’ was based on presence in plots where soil pH < 5 and C:N ratio exceeded 21. There were 14 alien taxa that met this definition of Tolerant: *Aira elegantissima*, *Anthoxanthum aristatum*, *Briza* spp., *Centaurium* spp., *Cirsium vulgare*, *Galium murale*, *Hypericum perforatum*, *Hypochaeris glabra*, *Hypochaeris radicata*, *Lysimachia arvensis*, *Sonchus asper*, *Sonchus oleraceus*, *Trifolium* spp., *Vulpia* spp. Extent of occurrence calculations as per Figure 2, main text

**FIGURE B3:**
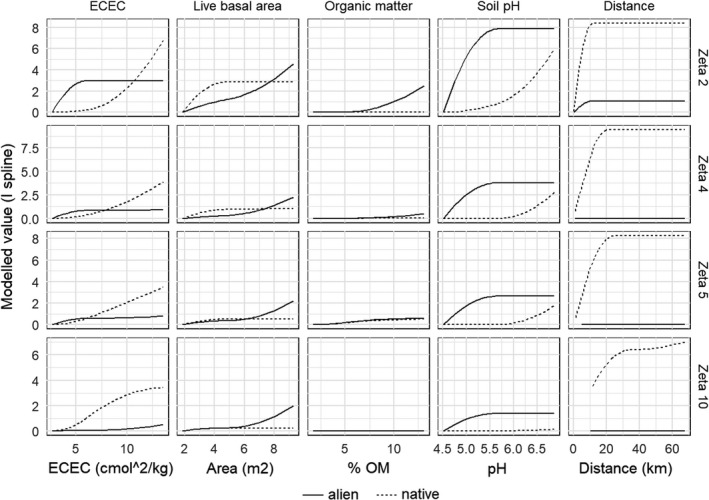
I spline functions for the four environmental variables and inter‐plot distance as a function of original scale range in the raw data. Rows show increasing order of zeta and therefore how the effects of the predictors change on species of increasing frequency of occupancy. Each column shows the effect for a single predictor as indicated above (ECEC = effective cation exchange capacity)

As expected, alien species turnover was better explained by environmental conditions than native species. Yet, despite the additional constraints on composition imposed by abiotic conditions (that might be expected to increase turnover from environmental spatial autocorrelation; Nekola & White, 1999), alien species turnover was much lower than that of native species (Figure 4). This pattern emerged because turnover in aliens was driven by a subset of species that were not only distributed widely but also tolerant of a wide range of environmental conditions (Figure B1, B2). The co‐occurrence of multiple tolerant species at most sites (albeit in different combinations) accounted for their lower overall turnover.

### 4.3 Annual life history of alien species might offer advantages

Alien and native species clearly fell predominantly within annual and perennial life histories, respectively. Many of the common and widespread alien species, including *Briza* spp., *Hypochaeris glabra*, and *Lysimachia arvensis*, were annuals, which often have traits that promote resource acquisition (van Kleunen et al., 2010). In the study region, alien species tend to have higher specific leaf area than natives (McGrannachan & McGeoch, 2019), promoting competitive dominance and possibly accounting for their preference for low carbon‐nitrogen ratio and higher soil pH sites, which represent more productive conditions. An annual life history also allows alien species to escape competition for resources with perennial native species during the low rainfall summer months when water becomes limiting (McGrannachan & McGeoch, 2019).

Annual species typically have large seed banks (Rees, 1993) and could be better placed to establish following natural mortality of longer‐lived perennial individuals or following disturbance, particularly fires. Notably, alien species were dominated by grasses such as *Aira elegantissima*, *Briza* spp., and *Vulpia* spp., which have the potential to increase fuel load, altering fire regimes (Brooks et al., 2004). While the role of fire in the spread of alien species was not examined here, alien propagules can outcompete natives during post‐fire regeneration even if local alien seed banks are reduced at the time of the fire (Keeley et al., 2005).

### 4.4 Implications and possible future trajectories in alien and native species

Although the scale of this study does not allow detailed analysis of coexistence, one of the questions raised by the results is the extent to which competition between the two components affects diversity and turnover. The negative alien‐native correlation in richness and the positive correlation in frequency suggest they are in direct competition – at least within the higher soil pH plots. Widespread alien species can out‐compete rare natives (Zhang & van Kleunen, 2019) and our data were consistent with greater competitive impact on rare native species at higher pH. It is possible this reflects an environmentally mediated influence of the alien and native components on one another. However, this would clearly require validation, either experimentally, or via time series monitoring of relative native and alien species performance at sites along a pH and productivity gradient. For example, future monitoring or experimental work could look to track changes in native species richness and relative abundance along a soil pH or C:N ratio gradient. Such an approach could also consider relative success in post‐fire establishment of native and alien species to guide any necessary management intervention to promote native plant biodiversity.

Alien plants were more widespread across the study region than natives, a pattern which has also been observed at the scale of biogeographical ranges of plants (Bradley et al., 2015). While this implies alien species are not as constrained as natives in their ability to reach and colonize sites, we are not able to determine whether this reflects superior germination, establishment success (Flores‐Moreno et al., 2013), higher propagule pressure from the surrounding landscape matrix, or the propensity for dispersal of herbaceous and graminoid propagules via anthropogenic activities (Pickering & Mount, 2010). It is possible that the generally more widespread distribution of (in particular) the more tolerant alien species will allow them to colonize unoccupied sites (Bradley et al., 2015), which would increase local alien richness and further decrease their turnover.

The incorporation of naturalized non‐native plants in community structure affects not only patterns of taxonomic diversity but can also impact functional and phylogenetic diversity (Sodhi et al., 2019; Vilà et al., 2011; Winter et al., 2009). Consistent with the patterns we found for turnover, the presence of alien understory species in this region has been associated with higher, but more homogeneous functional diversity (McGrannachan & McGeoch, 2019), along with stronger phylogenetic clustering (McGrannachan et al., 2020). Having become part of the spatial and temporal dynamics of local biodiversity, there is a risk that long‐term trajectories in community composition could shift toward increasingly alien‐dominated habitats (Catford et al., 2012; Jauni et al., 2015), impacting on taxonomic, functional, and phylogenetic diversity.

## 5 CONCLUSION

The ecological impact of multispecies introductions is still poorly understood, and here we found contrasting correlates for the diversity and turnover of rare and common alien and native species. Pressure from multispecies introductions in this semi‐natural dry forest understory ecosystem appears to be most acute under conditions of more neutral pH and higher productivity. Such sites could face greater risk for local loss of native diversity, consistent with the indirect finding of lower richness of rare native species at more neutral pH conditions. This suggests a possible composite impact of multiple introduced species, not all of which are necessarily individually considered problematic invasives (McGrannachan & McGeoch, 2019).

## CONFLICT OF INTEREST

The authors declare no conflict of interest.

## AUTHOR CONTRIBUTIONS

**Sarah Reeve:** Conceptualization (equal); Data curation (equal); Formal analysis (lead); Investigation (equal); Methodology (equal); Resources (supporting); Validation (equal); Visualization (lead); Writing – original draft (lead); Writing – review & editing (lead). **David C. Deane:** Formal analysis (supporting); Methodology (supporting); Writing – review & editing (lead). **Chris McGrannachan:** Data curation (equal); Investigation (equal); Validation (equal); Writing – review & editing (supporting). **Gillis Horner:** Data curation (supporting); Investigation (equal); Validation (equal); Writing – review & editing (supporting). **Cang Hui:** Conceptualization (equal); Formal analysis (supporting); Funding acquisition (supporting); Methodology (equal); Writing – review & editing (supporting). **Melodie A. McGeoch:** Conceptualization (equal); Formal analysis (supporting); Funding acquisition (lead); Investigation (equal); Methodology (equal); Project administration (lead); Resources (lead); Supervision (lead); Validation (equal); Visualization (supporting); Writing – original draft (supporting); Writing – review & editing (supporting).

## Data Availability

Data used in this analysis are archived with DOI accession number: 10.5281/zenodo.6216484 and code required to run the analysis is archived at github: https://github.com/deaned01/Diversity‐and‐Multispecies‐Invasion.

## APPENDIX A

### Additional background information

**TABLE A1:** Characteristics of each park within the study area: Chiltern Box‐Ironbark National Park, Mt Pilot Park, Baranduda Regional Park and Warby Ovens National Park, all of which are within Victoria, Australia

Park	Bioregion	Ecological Vegetation Classes	Park size (ha)	Disturbance History	Elevation range (asl)
Chiltern Box‐Ironbark National Park and Mt Pilot Park	Northern Inland Slopes	Alluvial Terraces Herb‐rich	21,560	Grazing present, fire recordings (from 1973 to 2010), erosion (present and absent from some sites)	198–572
		Box Ironbark Forest			
		Grassy Dry Forest			
		Granitic Hills Woodland			
		Heathy Dry Forest			
		Valley Grassy Forest			
Baranduda Regional Park	Northern Inland Slopes	Grassy Dry Forest	3,847	Grazing present, fire recordings (from 1940 to 2013), no erosion recorded	437–479
		Herb‐rich Foothill Forest			
Warby Ovens National Park	Northern Inland Slopes	Box Ironbark Forest	14,655	Grazing present, no fire history, no erosion recorded	170–371
		Grassy Dry Forest			
		Valley Grassy Forest			

**TABLE A2:** Summary statistics of the abiotic variables taken from the field across 2013–2015

Environmental Variable	Minimum	Mean (± SD)	Maximum
Carbon Nitrogen Ratio (C:N)	12.45	20.90 (± 4.90)	31.10
Effective Cation Exchange Capacity (ECEC)^a^	2.66	6.35 (±2.26)	13.56
pH^a^	4.49	NA	6.87
Organic matter^a^	1.68	5.68 (±2.48)	13.14
Altitude	170.00	371.37 (± 121.38)	572.00
Leaf‐area‐index	0.48	0.89 (± 0.21)	1.43
Live‐basal‐area^a^	1.88	5.07 (± 1.72)	9.29
Openness	25.08	37.48 (± 6.41)	51.45
Slope	0.00	3.55 (± 3.80)	17.50
Ammonium (Nitrogen)	2.97	10.07 (± 4.43)	19.92
Calcium	93.40	717.57 (± 453.48)	2064.89
Carbon	0.96	3.25 (± 1.42)	7.51
Conductivity	0.02	0.04 (± 0.02)	0.12
Hydration	0.00	4.33 (± 3.91)	16.32
Magnesium	45.09	159.42 (± 76.31)	401.12
Nitrate (Nitrogen)	0.85	1.63 (± 0.40)	2.72
Nitrogen	0.07	0.15 (± 0.05)	0.28
Phosphorus	1.17	2.48 (± 0.91)	5.01
Potassium	96.26	181.35 (± 59.33)	453.99
Sodium	4.40	15.23 (± 7.29)	43.66

^a^ Indicates variables used within the plant distribution analysis.

**TABLE A3:** Temperature (°C) data taken from the Wangaratta weather station (Australian Bureau of Meteorology) over the field seasons across 2013–2017

Month	2013^a^	2014^a^	2015^a^	2016^a^	2017
*July*					
Mean (± SD)					7.1 (± 6.3)
Max					−5.6
Min					16.2
*September*					
Mean (± SD)		11.3 (± 8.2)	10.5 (± 8.3)		
Max		−1.0	−1.5		
Min		24.1	23.6		
*October*					
Mean (± SD)	5.2 (± 8.9)	15.2 (± 10.0)	17.1 (± 10.1)		
Max	−1.7	1.5	−0.1		
Min	31	33.0	34.5		
*November*					
Mean (± SD)	16.1 (± 9.9)	18.5 (± 10.4)	19.0 (± 9.3)	17.3 (± 9.1)	
Max	0.6	0.2	1.8	5.2	
Min	33.6	34.9	37.4	36.1	
*December*					
Mean (± SD)	21.0 (± 10.8)	21.0 (± 9.5)	22.1 (± 10.4)		
Max	4.7	6.2	5.7		
Min	41.7	35.6	42.2		

^a^ Indicates years when plant surveys were conducted, ± indicates standard deviation from the mean.

## APPENDIX B

### Post hoc supporting analyses

**TABLE B1:** Summary statistics for the overall plant community (All plants) and the alien and native components at a plot scale (500 m^2^)

Data type	All plants	Alien component	Native component
*Incidence (presence or absence among plots)*			
Min	39	6	22
Mean (± SD)	59.7 (± 8.7)	19.4 (± 8.2)	40.2 (± 8.6)
Max	78	34	57
*Prevalence (frequency within plots)*			
Min	289	40	151
Mean (± SD)	648 (± 120.9)	219 (± 110.1)	429 (± 116.1)
Max	837	432	675

**TABLE B2:** Twenty most commonly occurring species across the 50 plots

Family	Species	Origin	Life history	Growth form	Plots occupied
Poaceae	*Briza* spp.	Alien	Short‐lived	Tufted graminoid	50
Araliaceae	*Hydrocotyle* spp.	Native	Short‐lived	Herb	50
Asteraceae	*Hypochaeris radicata*	Alien	Short‐lived	Herb	50
Poaceae	*Microlaena stipoides*	Native	Long‐lived	Rhizomatous graminoid	49
Primulaceae	*Lysimachia arvensis*	Alien	Short‐lived	Herb	48
Poaceae	*Vulpia* spp.	Alien	Short‐lived	Tufted graminoid	48
Haloragaceae	*Gonocarpus tetragynus*	Native	Long‐lived	Herb	47
Poaceae	*Rytidosperma* spp.	Native	Long‐lived	Tufted graminoid	47
Apiaceae	*Daucus glochidiatus*	Native	Short‐lived	Herb	46
Asparagaceae	*Lomandra filiformis*	Native	Long‐lived	Tufted graminoid	46
Oxalidaceae	*Oxalis perennans*	Native	Short‐lived	Herb	46
Asteraceae	*Senecio* spp.	Native	Long‐lived	Herb	46
Poaceae	*Aira elegantissima*	Alien	Short‐lived	Tufted graminoid	45
Geraniaceae	*Geranium* spp.	Native	Long‐lived	Herb	44
Asteraceae	*Hypochaeris glabra*	Alien	Short‐lived	Herb	44
Hypericaceae	*Hypericum gramineum*	Native	Long‐lived	Herb	42
Phyllanthaceae	*Poranthera microphylla*	Native	Short‐lived	Herb	42
Pteridaceae	*Cheilanthes austrotenuifolia*	Native	Long‐lived	Fern	40
Campanulaceae	*Wahlenbergia* spp.	Native	Long‐lived	Herb	40
Orchidaceae	*Microtis* spp.	Native	Long‐lived	Herb	39

**TABLE B3:** Twenty least prevalent species (observed in only 1 subplot)

Species	Origin	Life history	Growth form
*Amphibromus macrorhinus*	Native	Long‐lived	Tufted graminoid
*Brachyscome perpusilla*	Native	Short‐lived	Herb
*Bursaria spinosa*	Native	Long‐lived	Shrub
*Coronidium scorpioides*	Native	Long‐lived	Herb
*Dipodium hamiltonianum*	Native	Long‐lived	Herb
*Dipodium punctatum*	Native	Long‐lived	Herb
*Epilobium* sp.	Native	Short‐lived	Herb
*Eriochilus cucullatus*	Native	Long‐lived	Herb
*Euphorbia peplus*	Alien	Long‐lived	Herb
*Indigofera australis*	Native	Long‐lived	Shrub
*Isotoma axillaris*	Native	Long‐lived	Herb
*Leptospermum continentale*	Native	Long‐lived	Shrub
*Linum trigynum*	Alien	Short‐lived	Herb
*Parietaria debilis*	Native	Short‐lived	Herb
*Pultenaea largiflorens*	Native	Long‐lived	Shrub
*Pultenaea platyphylla*	Native	Long‐lived	Shrub
*Ranunculus parviflorus*	Alien	Short‐lived	Herb
*Sagina apetala*	Alien	Short‐lived	Herb
*Siloxerus multiflorus*	Native	Short‐lived	Herb
*Tripogonella loliiformis*	Native	Long‐lived	Tufted graminoid

**TABLE B4:** Independent and joint variance explained by distance and environmental variables (organic matter, live basal area, effective cation exchange capacity and soil pH) for the I‐spline based MS‐GDMs for the native and alien components shown in Figure 5, main text. Zeta order refers to the number of sites being combined and larger values represent the influence on more common species

Component	Zeta order	Distance	Dist:Env	Environment	Unexplained
Alien	2	0.002	0.007	0.303	0.688
	4	0	0	0.325	0.675
	5	0	0	0.319	0.682
	10	0	0	0.317	0.684
Native	2	0.118	0.041	0.123	0.718
	4	0.085	0.033	0.094	0.788
	5	0.064	0.030	0.085	0.822
	10	0.035	0.013	0.032	0.919
